# Developing electronic health records as a source of real-world data for veterinary pharmacoepidemiology

**DOI:** 10.3389/fvets.2025.1550468

**Published:** 2025-04-01

**Authors:** Heather Davies, Peter-John Noble, Ivo S. Fins, Gina Pinchbeck, David Singleton, Munir Pirmohamed, David Killick

**Affiliations:** ^1^Institute of Infection, Veterinary and Ecological Sciences, University of Liverpool, Liverpool, United Kingdom; ^2^Institute of Systems, Molecular and Integrative Biology, University of Liverpool, Liverpool, United Kingdom

**Keywords:** pharmacoepidemiology, electronic health records, text mining, adverse events, real world data, real world evidence

## Abstract

Spontaneous reporting of adverse events (AEs) by veterinary professionals and the public is the cornerstone of post-marketing safety surveillance for veterinary medicinal products (VMPs). However, studies suggest that most veterinary AEs remain unreported. Veterinary medicine regulators, including the United Kingdom Veterinary Medicines Directorate and the European Medicines Agency, have included the exploration of big data utilization to support pharmacovigilance efforts in their regulatory strategies. In this study, we describe the application of veterinary electronic healthcare records (EHRs) from the SAVSNET veterinary first opinion informatics system to conduct pharmacoepidemiological analyses. Five VMP-AE pairs were selected for investigation in a proof-of-concept study, where drug exposure was identified from semi-structured treatment data and AEs from the unstructured free-text clinical narrative. Dictionaries were developed to identify AEs based on standard terminology. The precision of these dictionaries improved when they were expanded using word vectorization and expert opinion. A key strength of first-opinion EHR datasets is their ability to enable cohort studies and facilitate calculations of absolute incidence and relative risk. Thus, we demonstrate that unstructured free-text clinical narratives can be used to identify outcomes for veterinary pharmacoepidemiological studies and, consequently, support and expand pharmacovigilance efforts based on spontaneous AE reports.

## 1 Introduction

Pharmacovigilance is defined as ‘the science and activities related to the detection, assessment, understanding, and prevention of adverse effects or any other drug-related problem’ (1). Central to this process is the spontaneous reporting of adverse events (AEs) by veterinary professionals and members of the public, either to the marketing authorization holder (MAH) or directly to the national competent authority (NCA). Despite the importance of AE reporting for the safety monitoring of veterinary medicinal products (VMPs), studies suggest significant underreporting (2–6). Therefore, exploring alternative data sources that can support and enhance pharmacovigilance efforts is warranted. Given this potential, regulatory agencies—including the European Medicines Agency and the United Kingdom’s NCA, the Veterinary Medicines Directorate (VMD)—have included the evaluation of big data approaches for pharmacovigilance in their regulatory strategies (7, 8).

In human healthcare, numerous studies utilize electronic healthcare records (EHRs) for pharmacovigilance (9, 10). A primary advantage of using EHRs for this purpose is the capability to calculate absolute incidence due to the availability of denominator information in the form of treatment data recorded in the EHR. In veterinary medicine, EHR-based drug safety studies have primarily depended on retrospective chart reviews of EHRs from single centers (11–13) or diagnosis codes assigned to EHRs to identify AEs when using large datasets from multiple centers (14–17). While these studies clearly demonstrate the value of veterinary EHRs for drug safety and efficacy research, manual retrospective chart review is labor-intensive, making scalability of this approach unappealing. Additionally, studies relying on diagnosis codes are constrained by the completeness and accuracy of the assigned codes.

The Small Animal Veterinary Surveillance Network (SAVSNET) is a veterinary informatics initiative created at the University of Liverpool that collects veterinary EHRs from participating first opinion practices in the United Kingdom. In addition to animal signalment data, SAVSNET collects treatment information and the free-text clinical narrative recorded by the veterinary professional during each consultation. A detailed account of the SAVSNET data collection process is available elsewhere (18). Previous work has been undertaken to map the treatment data captured by SAVSNET to active substances (19). Using this work, it is therefore possible to identify specific drug exposures for individual animals. Developing a method to detect AEs in the free-text clinical narrative would allow for the use of SAVSNET EHRs in pharmacoepidemiological analyses.

Therefore, the aim of this study was to create a method for identifying VMP-AE pairs in SAVSNET EHRs. We utilized five example VMP-AE pairs as a proof of concept. Accordingly, the specific objectives were:

1. To identify cohorts of animals based on their exposure to specific VMPs.
2. To develop dictionaries of terms related to the AEs of interest, facilitating the identification of mentions of these AEs in SAVSNET EHRs.
3. To use data from objectives one and two to calculate the absolute incidence of the AEs of interest and relative risk compared to a comparator VMP.

## 2 Materials and methods

### 2.1 Ethics

The SAVSNET project has received ethical approval from the University of Liverpool Research Ethics Committee (RETH001081).

### 2.2 Drug-event pair selection

Five VMP-AE combinations, representing four VMPs and four AEs, were selected in consultation with the VMD. These VMP-AE pairs had previously been identified for further monitoring by the European Medicines Agency. As this is a proof-of-concept study, the integration of results into signal management and pharmacovigilance risk mitigation strategies has not been explored. Therefore, the VMP names have been concealed, and the VMPs are referred to using the labels ‘VMP-A,’ ‘VMP-B,’ ‘VMP-C,’ and ‘VMP-D.’ However, it is important to note that the outcomes have been shared with the VMD. The VMP-AE pairs were as follows:

1. VMP A-Blindness
2. VMP B-Convulsions
3. VMP C-Hepatopathy
4. VMP D-Renal insufficiency
5. VMP C-Convulsions

All of these examples pertain to dogs, except for VMP A-Blindness, which focuses on cats. One comparator VMP was selected for each of the VMPs of interest. Comparators were chosen with advice from veterinary surgeons alongside the Summaries of Product Characteristics (SmPC). Comparator VMPs had the same or similar indications as the VMP of interest and were free from any known or suspected risk of the AE of interest, except for the comparator selected for VMP-D, where the AE of interest represents a known pharmaceutical class effect.

Case definitions for the AEs of interest were developed with the clinical expertise of domain experts (veterinary surgeons practicing in an academic referral center, one having additional experience in drug safety and the other in veterinary health informatics) and are shown in Table 1.

**Table 1:** Case definitions for the AEs of interest.

Adverse event	Case definition
Visual impairment/blindness	The clinical narrative contains a clear indication of limited/deteriorating vision or blindness as determined by a veterinary professional OR Clear description of the patient showing behavior consistent with blindness (e.g., bumping into things, does not track cotton ball) OR A clinical narrative includes descriptions of abnormal visual reflexes/responses or pathological anatomical alterations
Convulsions	The clinical narrative contains a clear indication that the animal has had a convulsion OR This is a clear indication that the veterinary professional suspects the animal has had a convulsion OR A clear description of the patient showing behavior consistent with convulsions, either focal or generalized (e.g., twitching muscles, jerking, paddling, drooling/foaming at mouth, vacancy, collapse, and/or loss of consciousness, nystagmus)
Hepatopathy	The clinical narrative indicates ALT is elevated and/or ALT is above (150 IU/L) OR Bile acids are elevated OR Veterinary professionals indicate that they suspect a hepatopathy OR Clear description of the patient presenting with jaundice in the absence of anemia OR The patient has been prescribed a liver disease-specific treatment such as Samylin®, Denamarin®, Destolit® or ursodeoxycholic acid
Renal insufficiency	Patients prescribed kidney disease-specific treatment such as a renal diet or Ipakitine® or Pronefra® OR The clinical narrative contains a statement indicating a diagnosis of kidney disease OR The clinical narrative reports a result consistent with a diagnosis of kidney disease (specific gravity <1.030, creatinine >125, SDMA >14, UPC > 0.5)

### 2.3 AE identification

To identify the AEs of interest in SAVSNET data, dictionaries containing words and phrases that may be used by veterinary professionals to record these AEs within the free-text clinical narrative portion of the EHR (referred to as ‘clinical narratives’ herein) were developed. The dictionaries were applied to SAVSNET data as regular expressions, an approach for identifying specific (and often complex) patterns within the text (20).

#### 2.3.1 Initial dictionary: VeDDRA term selection

The Veterinary Dictionary for Drug Regulatory Activities (VeDDRA) version 15.0 was utilized to develop the initial dictionary for each AE. A comprehensive top-down examination of VeDDRA was performed for each AE, commencing with the pertinent system organ class, to identify VeDDRA codes for potential inclusion. The VeDDRA ‘lower-level’ terms (LLTs) were selected, as this category most accurately reflects the terminology that may be employed to articulate individual clinical signs and symptoms in a clinical context, as opposed to the ‘preferred term’ (PT) category, which encompasses broader medical concepts. A regular expression was created using the VeDDRA LLTs contained within these dictionaries.

#### 2.3.2 Expanded dictionary: word vectorization model training

Recognizing that the VeDDRA regulatory language is unlikely to cover the full range of terminology used in clinical narratives, the initial dictionaries were expanded using a word vectorization approach to identify corpus-specific synonyms, misspellings, and abbreviations. A random sample of 1,000,000 SAVSNET clinical narratives was used to train a word vectorization model (word2vec) using the Gensim Python library (21). The Skip-gram iteration was chosen over the continuous bag-of-words approach due to the superiority of skip-gram in capturing the semantics of words (22).

Narrative pre-processing involved splitting each narrative into separate sentences, removing capitalization, tokenization using the Natural Language Toolkit (NLTK) package (23), and removing punctuation via regular expression substitution. Finally, bigrams were generated using Gensim’s Phrases function.

A series of models was generated to investigate the optimal values for the vector size, window size, and minimum count parameters, in turn. All other parameters were set to their default values, and the number of iterations was fixed at 15. The similar_terms function takes an input word and provides an output of terms within the corpus along with their cosine similarity to the input word, starting with the highest cosine value (i.e., the most similar to the input word). This function was used to determine the optimal value, using ‘diarrhoea’ as the input word. Diarrhoea was chosen due to the numerous misspellings, abbreviations, and synonyms possible within free text. For each model, the output of the 30 most similar words and phrases was reviewed, and the parameter value that yielded the most suggestions for misspellings, abbreviations, and synonyms with the fewest irrelevant terms was selected. When there was little difference in the performance of the various models, the original word2vec work (22) and the application of word2vec for a similar task (24) were referenced to guide the final model choice.

As the similar_terms function returns a cosine similarity value for each token within the corpus, a method was needed to determine how many of these terms should be manually reviewed for inclusion in the expanded dictionary. Instead of specifying an arbitrary number of terms for review, which risks overlooking relevant terms when there are numerous tokens with high similarity, a cut-off cosine similarity value was established for each target word. Hypothesizing that the VeDDRA PT (i.e., the broad medical concept) would have a similar cut-off value to the individual LLTs, we used each of these PTs as the input word to generate individual outputs for review. Each output was reviewed, starting with the highest cosine similarity, until no new misspellings or unique synonyms were identified within 10 consecutive terms. The cosine similarity value for the last relevant term was then selected as the cut-off.

The similar_terms function was used to generate an output of similar words and phrases with a cosine similarity greater than or equal to the determined cut-off value for each of the VeDDRA LLTs included in the initial dictionary. One reviewer reviewed the outputs to create an expanded dictionary, which was then used to develop an expanded regular expression. Where applicable, word stems were utilized to incorporate multiple individual terms into the regular expression.

#### 2.3.3 Regular expression development

Regular expression development was conducted iteratively by testing the regular expression on a random sample of clinical narratives and reviewing the matches to identify false positives. After each test, the regular expression was updated. For example, the convulsions regular expression was refined to ensure the phrase ‘fit and well’ was ignored while still capturing mentions of ‘fit,’ which may be used to describe convulsions. A new random sample was used following each update. In the absence of a formalized benchmark, this process was repeated until the reviewer felt that false positives had been minimized sufficiently.

#### 2.3.4 Regular expression finalization: expert opinion

The final stage in developing the regular expressions involved an expert review. Domain experts—both veterinary surgeons practicing in an academic referral center, one with additional experience in drug safety and the other with additional expertise in veterinary health informatics—examined each of the final dictionaries and regular expressions, suggesting updates. Notably, they recommended that the regular expressions for renal insufficiency and hepatopathy include terms to capture relevant test results. These suggestions were incorporated into the regular expressions, and further iterative testing was conducted to ensure they did not lead to additional false positives. Following this process, the regular expressions were deemed final. The final regular expressions are available in the Supplementary materials S1.

#### 2.3.5 Regular expression precision

A random sample of 10,000 clinical narratives was used to assess the precision of the regular expressions. Two independent reviewers evaluated matches against the case definitions (Table 1). Precision was determined by dividing the number of reviewer-confirmed cases by the total number of matches. Due to the presence of a gold standard dataset containing practitioner-confirmed cases of renal insufficiency, the recall of the renal insufficiency regular expression was also evaluated by dividing the total number of matches by the total number of true positives.

### 2.4 Exposure identification

Animals prescribed the VMP of interest or the comparator VMP were identified using a previously classified dataset (19). Within this dataset, product descriptions are mapped to standardized active substances. The dataset was filtered by active substance to create a list of unique animals that had received the VMP of interest. This list identified the initial prescribing event (i.e., index prescription) for each animal. Further filtering was conducted to remove animals receiving a different dosage form than the VMP of interest. Dosage form is not a standardized field in this dataset; therefore, this task was carried out using manual filtering within Microsoft Excel (2016) and regular expression-based matching of the raw product description. Animals were removed from the dataset for prescription events where multiple dosage forms exist if the specific VMP could not be identified.

The data lock point of this pre-mapped dataset was 24-Feb-2020. Therefore, only animals with an initial prescribing event occurring between the start of SAVSNET data collection and 24-Feb-2020 were included in each cohort.

**Figure 1:**
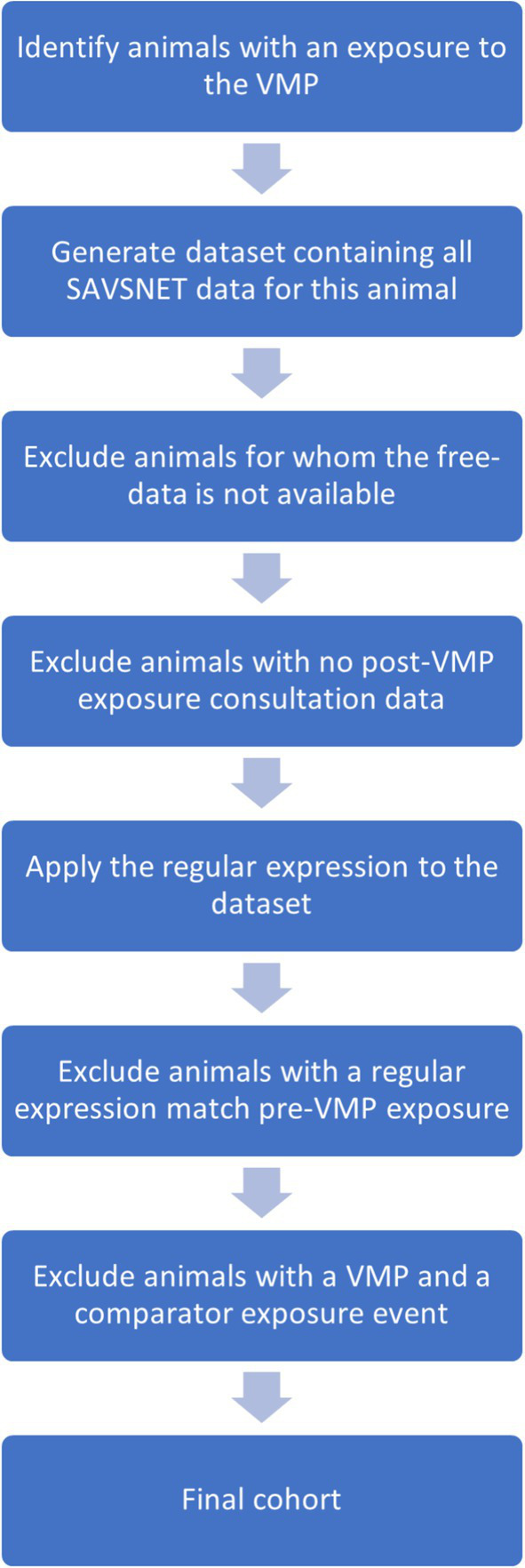
An overview of the process used to define the final cohort of exposed animals, beginning with the identification of animals that had exposure to the VMP of interest and detailing each stage of exclusions.

For the remaining animals, the unique SAVSNET ID was used to generate a dataset that included each animal’s complete SAVSNET consultation history (i.e., pre- and post-drug exposure). Note that the data lock point mentioned earlier was not applied during this stage. Animals without post-exposure data were excluded. The finalized regular expressions were applied to the pre-drug exposure consultations, and any animals with the outcome of interest occurring before drug exposure were removed. Finally, any animals appearing in both cohorts (i.e., VMP of interest and comparator VMP) were eliminated to prevent contamination between groups. An overview of this process is shown in Figure 1.

### 2.5 Follow-up period identification

Previously submitted AE reports and pharmacokinetic data found in SmPCs were used to establish a post-exposure period during which an AE was likely to occur. In this analysis, we calculated the median time-to-onset of the AE and the median absolute deviation for the previously submitted AE reports. If the drug half-life indicated that the drug would remain in the system longer than the calculated median time-to-onset, then the equivalent of five half-lives (i.e., estimated total elimination) was applied. In the final dataset, animal histories were limited to include only post-exposure consultations within the following time periods:

- VMP A-blindness = 28 days
- VMP B-convulsions = 50 days
- VMP C-hepatopathy = 14 days
- VMP D-renal insufficiency = 52 days
- VMP C-convulsions = 50 days

### 2.6 Analysis

Finalized regular expressions were applied to the VMP-exposed and comparator VMP final datasets. A rule-based approach was then used for the AEs related to renal insufficiency and hepatopathy to eliminate matches associated with normal test results. All other matches were deemed to be cases.

Incidence was calculated by using the total number of identified cases as the numerator and the total number of animals in the cohort as the denominator, expressed per 10,000 animals. Relative risk and 95% confidence intervals (CIs) were calculated for each VMP/comparator VMP-AE pair.

All analyses were conducted using Python (version 3.7.0) in Jupyter Notebook, utilizing the pandas (25) and SciPy (26) packages.

Given that renal-related AEs are a known pharmaceutical class effect for the group of products to which VMP-D belongs, we conducted further analyses to examine the baseline exposure of animals in the VMP-D and comparator VMP cohorts to drugs within the same class. It was not feasible to calculate total exposure due to the nature of the available data (i.e., invoicing data missing the total quantity of tablets or volume of liquid dispensed and/or lack of generated prescription labels). Therefore, exposure was determined based on one prescription event equating to one exposure. We controlled for age confounding in these cohorts using the Mantel–Haenszel method to calculate an age-adjusted relative risk using the metafor (27) package in R (version 4.1.2).

## 3 Results

### 3.1 Dictionary expansion

The final word2vec parameters chosen were a vector size of 300, a window size of five, and a minimum count of eight. Using this model, an additional 59 terms were identified for the hepatopathy dictionary, 35 for renal insufficiency, 37 for blindness, and 106 for convulsions. Notably, some of these terms are included in the final regular expression in stemmed format, while others were not included at all following expert review or due to the number of false positives introduced, as identified during testing. For example, the terms ‘epileptic,’ ‘epileptic type,’ and ‘epileptiform’ were stemmed to ‘epilep’.

### 3.2 Regular expression validation

Generally, the precision of the regular expressions improved with the expansion of the dictionaries using word embeddings and expert opinion. For the renal insufficiency regular expression, recall was also improved following this expansion. The precision and recall (where available) for the three dictionaries developed for each AE are shown in Table 2. Further investigation was warranted due to the low precision of the renal insufficiency regular expression. Therefore, the regular expression was divided into three distinct parts: mentions of renal disease, test results, and references to renal diet or treatment. Testing the three parts separately revealed that precision was highest when considering only mentions of renal diet or treatment (0.8), compared to those referring to renal disease (0.35) and test results (0.44). Nevertheless, all three parts were retained since recall was greatest (0.68) when all segments were included, compared to mentions of renal disease (0.38), test results (0.16), or renal diet or treatment (0.42) alone.

**Table 2:** Precision and recall for regular expressions based on three different dictionaries.

Adverse event of interest	Regular expression	Precision	Recall
Blindness/impaired vision	Initial dictionary	0.67	N/A
	Expanded dictionary	0.72	
	Final dictionary	1.00	
Convulsions	Initial dictionary	0.18	N/A
	Expanded dictionary	0.27	
	Final dictionary	0.47	
Hepatopathy	Initial dictionary	0.67	N/A
	Expanded dictionary	0.24	
	Final dictionary	0.61	
Renal insufficiency	Initial dictionary	0.17	0.07
	Expanded dictionary	0.27	0.52
	Final dictionary	0.43	0.68

Initial dictionary, based on VeDDRA terms alone; Expanded dictionary, expanded using word embeddings; Final dictionary, expanded further using expert opinion.

### 3.3 Exposure identification

Across the VMP cohorts, the number of animals receiving the VMP ranged from 1,403 to 34,503. After exclusions, these cohorts ranged from 503 to 23,558. Overall, the primary reason for exclusion was the lack of post-exposure consultation data. The total number of unique animals identified with a documented exposure event for each of the VMPs of interest, as well as for the comparator VMPs and exclusions, is shown in Table 3. Notably, for consultations occurring at practices using one specific practice management system, the free-text clinical narratives are not available in the SAVSNET database. These consultations are shown in the ‘data unavailable’ column.

**Table 3:** Number of unique animals with a recorded exposure to each product and comparator, the number of animals excluded at each step, and the number of animals in each of the final cohorts.

		Index prescriptions	Data unavailable	No post-exposure data	Pre-existing clinical signs	Product crossover	Final cohort
Product A	Blindness	11,158	2,980	2,789	15	20	5,354
Comparator A		101,717	304	24,357	208	20	76,828
Product B	Convulsions	34,503	4,904	5,052	738	251	23,558
Comparator B		1,571	162	235	35	251	888
Product C	Hepatopathy	12,736	2,136	2,496	24	1,377	6,703
Comparator C		287,343	12,777	60,635	449	1,377	212,105
Product D	Renal insufficiency	1,403	175	312	81	305	530
Comparator D		249,276	33,944	50,355	3,731	305	160,941
Product C	Convulsions	12,736	2,136	2,496	171	1,340	6,593
Comparator C		287,343	12,777	60,635	2,962	1,340	209,629

### 3.4 Outcome identification

AEs were identified within the post-exposure window for all cohorts except the VMP-C cohort. Table 4 shows the number of cases (i.e., animals with a post-VMP exposure regular expression match), the absolute incidence rate per 10,000 animals, relative risk (RR), and 95% CI. Due to the lack of AEs identified for this pair, no further analysis was performed to investigate the relative risk for hepatopathy among animals receiving VMP-C or comparator VMP.

**Table 4:** Number of cases in each product and comparator cohort, with the calculated absolute incidence per 10,000 animals and the corresponding relative risk (RR) and 95% CIs.

	Blindness	No signs	Total	Incidence (per 10,000 animals)	RR	95% CI
Product A	1	5,353	5,354	1.9	0.80	0.11–5.97
Comparator A	18	76,810	76,828	2.3		

	Convulsions	No signs	Total	Incidence	RR	95% CI
Product B	79	23,479	23,558	33.5	1.49	0.37–6.05
Comparator B	2	886	888	22.5		

	Renal signs	No signs	Total	Incidence	RR	95% CI
Product D	8	522	530	150.9	2.87	1.44–5.7
Comparator D	847	160,094	160,941	52.6		

	Convulsions	No signs	Total	Incidence	RR	95% CI
Product C	18	6,575	6,593	27.3	1.33	0.82–2.09
Comparator C	438	209,191	209,629	20.9		

There was a greater risk of developing renal insufficiency within a 52-day window following exposure to VMP-D compared to the comparator product (RR = 2.87, 95% CI 1.44–2.57). Using the information available in the EHRs, we were able to conduct further analysis. Overall, animals receiving VMP-D had a greater number of prior prescriptions for VMPs in the same pharmaceutical class compared to animals in the comparator cohort (1.27 prescriptions per 1,000 animal days versus 0.04 prescriptions per 1,000 animal days). However, the cases from the VMP-D cohort had received a similar number of prior prescriptions when compared with non-cases (1.34 vs. 1.27 per 1,000 animal days). When analyzing by age, the relative risk calculated for each stratum was as follows: <10 years RR = 1.49, 95% CI 0.21–10.56, and > 10 years RR = 1.92, 95% CI 0.92–4.01. The age-adjusted relative risk was determined to be 1.85 (95% CI 0.93–3.69), suggesting that further evaluation as a larger cohort becomes available is necessary.

## 4 Discussion

This study demonstrates for the first time that valuable pharmacoepidemiological data can be obtained from large, unstructured first-opinion EHR datasets in the veterinary context. Furthermore, we have outlined a method for identifying cohorts of animals based on their exposure to a VMP and for identifying mentions of specific clinical signs representative of five AEs of interest. Together, these two steps facilitate the identification of VMP-AE pairs in veterinary EHRs for pharmacoepidemiological studies. Specifically, we show that free-text data can be utilized for AE identification, which can be further enhanced with the accompanying structured and semi-structured data from the EHR, such as drug exposure and animal signalment information. Thus, data produced by this approach is potentially useful to NCAs and MAHs, as well as to clinicians making risk–benefit prescribing decisions, since it enables the estimation of risk at a per-patient level and allows for comparisons of those risks to a within-indication comparator product.

EHRs are widely used in human healthcare for identifying AEs, with several large databases supporting this effort, including the Clinical Practice Research Datalink (CPRD) in the United Kingdom (28) and the Sentinel initiative in the US (29). In fact, a previous study showed that an acute myocardial infarction signal could be detected using the EU-ADR network of EHR databases four years prior to its identification through traditional data sources (30).

The digitization of records and the databases of EHRs, curated through projects such as SAVSNET (18), VetCompass (31), and the Banfield group (32), means that substantial volumes of EHRs are accessible for epidemiological research in veterinary medicine. Our previous work suggests that veterinary AEs are frequently documented within EHRs, even when they are not formally reported (33).

Studies identifying veterinary AEs in large datasets have mostly relied on diagnosis codes (14–17). In these studies, a list of relevant diagnosis codes is outlined at the outset, and these codes are then used to identify cases of interest within the databases. In this regard, AE detection depends on the complete and accurate coding of every consultation present in the EHR. In human medicine, it has been shown that there are several obstacles to achieving high inter-annotator agreement when coding EHRs, even among medically trained annotators (34). This issue is not well explored in veterinary medicine, partly because manual annotation of entire datasets is not common clinical practice. However, given the lack of a standardized approach to coding or a universally accepted coding framework, similar challenges likely exist.

Identifying AEs in free text would avoid the problems associated with non-systematic data coding. Since it is not feasible to manually screen tens of thousands to millions of records, big-data approaches are necessary to identify cases of interest. Here, we demonstrated that VeDDRA can serve as a foundational ontology for AE identification and that the precision of subsequent searches is improved by expanding VeDDRA terminology with misspellings and synonyms identified through a word embeddings approach. Notably, precision improved further after review by a domain expert (a veterinary surgeon), emphasizing the importance of including experts in developing the search strategy. Word2vec has been successfully employed in other studies. In one study, word2vec was utilized to expand a dictionary of dietary supplements, resulting in an 8.3% increase in recall (35). Additionally, another study used word2vec to enlarge a dictionary of known AE terms, finding that the resulting AE rates were more accurate with the expanded dictionary than with the original (24).

We tested the precision of each developed regular expression on a random sample of 10,000 clinical narratives. The precision of the regular expressions for convulsions and renal insufficiency was found to be low, at 0.47 and 0.43, respectively. This was somewhat expected for the convulsions regular expression due to the inclusion of the term ‘fit,’ which is frequently used to describe convulsions but can also be employed in various other contexts. We attempted to control for this by specifying many of these contexts in the regular expression using negative lookbehinds and negative lookaheads. These two assertions mean that a dictionary term is only matched if it is not preceded or followed by other specified terms. For instance, the negative lookbehind ‘(? <!good/s)fit’ ensures that the sentence ‘harness is a good fit’ is not matched, while the negative lookahead ‘fit/s(?!and well)’ ensures that ‘dog is fit and well’ is not matched. However, it is not feasible to define all the ways in which the word ‘fit’ is used in a sentence in unstructured clinical narratives. Consequently, several of these instances were missed, contributing to the poor precision of the regular expression. We also found that the regular expression for hepatopathy did not follow the pattern of increasing precision as the dictionaries were updated. We suspect this is due to the numerous abbreviations for the various liver evaluative test indicators (e.g., ALT, ALP) included in the final regular expression. We chose to include these terms despite their minor negative effect on overall precision, as we hypothesized that they might improve recall of potential cases due to the high likelihood that liver-related tests would be performed and discussed prior to a definitive diagnosis.

The primary advantage of using a word embeddings approach is that the identified synonyms and misspellings are specific to the corpus. This provides flexibility when working with various datasets (for example, data derived from different practice groups, regions, or settings), as dictionaries can be tailored for each corpus. A drawback is that model generation and optimization can be time-consuming, although GPU-equipped systems have significantly reduced the time required to complete these tasks. Another aspect to consider is that models may need periodic updates due to the constantly evolving nature of natural language. Given how the similar_terms function operates (i.e., a target word is provided, and an output is generated consisting of *n* terms with the closest cosine similarity), the target word must appear in the training set more frequently than the threshold defined as the ‘minimum count’ parameter. In this case, the minimum count was eight, and while this did not appear to influence the number of new relevant terms identified during the model testing process, some less common terms were likely excluded. Therefore, this approach may not be suitable for developing dictionaries for rare events or those described in a pathognomonic manner, especially if the training set is not adequately large.

Large language models (LLMs) offer an alternative approach to AE identification and have garnered significant interest in human pharmacovigilance. These models are trained on extensive datasets and, therefore, eliminate the need to develop dictionaries of terms for detection. Various models have been tested, including GPT and BERT-based variants, yielding promising results. A fine-tuned model, AE-GPT (a GPT 3.5 model), achieved an F1 score of 0.70 for detecting vaccine AEs from spontaneous reporting data (36). Studies have also investigated using social media data as a source of AEs. Social media data may be more closely related to unstructured clinical data than traditional AE reports due to the likelihood of misspellings, abbreviations, and informal language styles. In these studies, fine-tuned BERT-based models were utilized, attaining F1 scores of 0.80 (37) and 0.86 (38). Based on the results of these studies, we propose further exploration of LLMs for AE identification from veterinary clinical narratives, and PetBERT, the BERT-based model fine-tuned on SAVSNET clinical data (39), provides an opportunity to do so.

Although not the primary purpose of this study, we conducted preliminary pharmacoepidemiological analyses of the VMP-AE pairs identified. Two of the VMP-AE pairs (VMP C-convulsions and VMP A-blindness) are listed in the respective SmPCs as known but very rare events. According to the rule of three, the cohort size required to detect these events would be 30,000 animals (40). Although the cohort sizes we identified are not sufficiently powered to detect very rare events, a benefit of using EHRs is that data is collected in near real-time, allowing studies to be repeated at regular intervals as cohort sizes increase. Additionally, to avoid potential contamination between the VMP and comparator cohorts, we excluded animals that had received both products in any order at any time in their SAVSNET history. This was necessary due to complexities in defining when an animal was considered to be ‘off-drug’ for a particular product. This presents a particular issue in veterinary medicine, where preventative medicines are sold in multiple pack sizes to be dosed at non-regular intervals (i.e., at intervals of a month or longer). Evidence suggests that these products are administered sporadically (41). Furthermore, these products can be purchased for one animal during a veterinary visit, but subsequent doses in the pack may be used to treat other animals in the household. An obvious limitation of this approach is that animals may experience an AE due to the VMP of interest and then subsequently change treatment to the comparator product, meaning that these AEs would not be detected.

Whilst the cohort for VMP-D was relatively small, we suspected that these animals were at a higher risk of renal insufficiency than those receiving the comparator product. The availability of other data within the EHR allowed us to explore this further. First, animals in the VMP-D cohort were older than those receiving the comparator product, increasing the likelihood that they were already experiencing age-related renal function decline. An age-adjusted relative risk of 1.85 suggests that the crude relative risk of 2.87 may have been inflated by an increased risk in older animals. However, this should be interpreted with caution given the 95% CI of 0.98–3.69. Further, while animals in the VMP-D cohort had a higher number of prior prescriptions for a drug in the same class at baseline compared to those in the comparator cohort, the same pattern was not observed when comparing cases and non-cases within the VMP-D cohort. Since testing does not generally identify renal dysfunction until there has been a significant loss of function (42), we suggest repeating this study later with a larger post-exposure window of interest to account for this.

Perhaps the most significant advantage of using EHRs for AE detection is the ability to calculate absolute incidence. Incidence values cannot be calculated from traditional pharmacovigilance data given that the number of reports (i.e., the numerator) does not take into account the known high levels of underreporting and the true number of exposed patients (i.e., the denominator) is unknown and therefore has to be estimated from sales data. The availability of exposure data in EHRs taken from prescription labels and invoicing information means that exposure can be more reliably calculated when compared to using sales data. However, there are some nuances worth discussing. First, prescription label information is likely incomplete for products administered to animals during consultations, as the product does not leave the premises. Since prescription labels usually provide more detailed descriptions of a product than the product dispensed information (which is primarily generated for invoicing), identifying the specific product formulation and dosage can be challenging when prescription label information is missing. Validation studies would be beneficial in fully understanding the extent of this issue. Secondly, animal owners are increasingly purchasing veterinary products online, which means that not all exposures are reflected in the EHR.

Additionally, unlike human healthcare systems, animals lack a unique personal identifier that accompanies them across various veterinary practices and settings. Animals may be presented to multiple different practices across their lifetime making it difficult to calculate total exposure at the individual level with certainty and resulting in ‘loss to follow-up’ in cohort-based studies. The method for identifying AEs discussed here relies on examining the clinical narrative for mentions of AEs; as a result, animals without post-exposure consultation data were excluded. Excluding these animals may have introduced bias into the study, potentially leading to either an under- or overestimation of the AE incidence reported here. First, it is possible that animals without consultation data following the initial exposure did not return for veterinary care because treatment was successful or because ongoing care was provided by a different veterinary practice. This scenario would result in an overestimation of AE incidence (on a per-regular-expression basis) if no AE occurred, particularly in cases where the sample size is small. On the other hand, there is a possibility that animals lost to follow-up experienced a serious AE, prompting the owner to seek urgent veterinary care from a different veterinary practitioner. This limitation is inherent to EHR-based companion animal health surveillance. An advantage of using a system like SAVSNET to investigate AEs is that data continually accumulates, and analysis can be repeated. Therefore, we suggest that the analysis for VMP-D be revisited after further data accumulation, especially since VMP-D is indicated for a chronic condition, making it likely that these animals may return for veterinary care. Furthermore, during the study period, it became a legal requirement for dogs and cats to have an identifying microchip. Thus, future research could develop methods for tracking animals (in this regulatory region) across practices using this number.

The methods outlined here depend on prior identification of a VMP-AE pair of interest and some knowledge of the likely post-exposure window during which an AE is expected to occur. In this regard, it represents an approach for signal validation (i.e., to support the investigation of AEs identified through existing pharmacovigilance methods). It is however, perhaps less useful for signal generation. Rapid advancements in machine learning and AI could prove essential for fully realizing the potential of EHRs in pharmacovigilance and pharmacoepidemiology. This approach could be enhanced by preparing a library of search terms for each VEDDRA term. Utilizing LLMs offers an exciting and potentially less labor-intensive opportunity to identify AEs in unstructured data by automating some of the steps described and detecting previously unknown AEs.

## 5 Conclusion and future direction

This study outlines that EHRs represent a rich data source that can be utilized to conduct pharmacoepidemiological analyses. Medicine regulators globally have highlighted the exploration of alternative data sources as a priority, and this study demonstrates that methods can be developed to use free-text clinical narratives to meet that need. The ability to calculate absolute incidence is a significant advantage of using EHRs, and can complement existing regulatory processes.

## Data Availability

Reasonable requests to access SAVSNET data can be submitted through an application process found at: www.liverpool.ac.uk/savsnet/using-savsnet-data-for-research/.
