# Supplementary material for: Developing electronic health records as a source of real-world data for veterinary pharmacoepidemiology
Source: Front Vet Sci. 2025 Apr 1;12:1550468. doi: 10.3389/fvets.2025.1550468 (PMC11996780; doi:10.3389/fvets.2025.1550468)
Supplement: Supplementary file 1 [file Data_Sheet_1.PDF]

frontiers

Supplementary Material

1 Supplementary Data

<table>
<tr><th>Event of interest</th><th>Finalised regular expression</th></tr>
<tr><td>Blindness</td><td>(?i)(?<=low\s)vision|(?<=limited\s)vision|(?<=flashing\s)vision|(?<=double\s)vision|(?<=blurred\s)vision|(?<=abnormal\s)vision|(?<=poor\s)vision|(?<=loss\sof\s)vision|(?<=impaired\s)vision|vision(?=\sblurr|\sdeteriorat|\sloss)|(?<!not\s)(?<!treat\s)(?<!treating\s)blind(?!\sfood|\streat)|(?<=negative\s)me?a?na?n?ce|(?<=-ve\s)me?a?na?n?ce|(?<=absent\s)me?a?na?n?ce|(?<=abnormal\s)me?a?na?n?ce|me?a?na?n?ce(?=\snegative|\sabsent|\s-ve|\sabnormal)|me?a?na?n?ce\sreflexe?s?(?=\snegative|\sabsent|\s-ve|\sabnormal)|me?a?na?n?ce\sresponc?s?es?(?=\snegative|\sabsent|\s-ve|\sabnormal)|(?<=negative\s)plr|(?<=negative\s)prl|(?<=negative\s)light\sreflex|(?<=negative\s)pupill?a?r?y?\s?l?i?g?h?t?\s?r?e?|(?<=-ve\s)plr|(?<=-ve\s)prl|(?<=-ve\s)light\sreflex|(?<=-ve\s)pupill?a?r?y?\s?l?i?g?h?t?\s?r?e?|(?<=absent\s)plr|(?<=absent\s)prl|(?<=absent\s)light\sreflex|(?<=absent\s)pupill?a?r?y?\s?l?i?g?h?t?\s?r?e?|(?<=abnormal\s)plr|(?<=abnormal\s)prl|(?<=abnormal\s)light\sreflex|(?<=abnormal\s)pupill?a?r?y?\s?l?i?g?h?t?\s?r?e?|(?<=sluggish\s)plr|(?<=sluggish\s)prl|(?<=sluggish\s)light\sreflex|(?<=sluggish\s)pupill?a?r?y?\s?l?i?g?h?t?\s?r?e?|plr(?=\snegative|\sabsent|\s-ve|\sabnormal|\ssluggish)|prl(?=\snegative|\sabsent|\s-ve|\sabnormal|\ssluggish)|light reflex(?=\snegative|\sabsent|\s-ve|\sabnormal|\ssluggish)|pupill?a?r?y?\s?l?i?g?h?t?\s?reflexe?s?(?=\snegative|\sabsent|\s-ve|\sabnormal|\ssluggish)|pupill?a?r?y?\s?l?i?g?h?t?\s?responc?s?es?(?=\snegative|\sabsent|\s-ve|\sabnormal|\ssluggish)|pupill?a?r?y?\slight(?=\snegative|\sabsent|\s-ve|\sabnormal|\ssluggish)|loosing\ssight|partiall?y?\ssight|poor\se?y?e?sight|e?y?e?sight\strouble|sight\saffected|bump\w{0,4}\sinto|bang\w{0,4}\sinto|wander|trip\w{0,4}\sover|diplopia|(?<!follows\s)cotton\sball|visual(?=\sfield|\sfield\sdefect|\simpairment)|tapetal\shyper-?r?eflectiv|difficulty\sfocusing|focusing\sdifficult|unable\sto\sfocus</td></tr>
<tr><td>Convulsions</td><td>(?i)convuls|epilep|rigid|(?<!no\s)(?<!not\s)si?e?i?zi?ur|(?<!no\s)(?<!not\s)seixure|tonic|clonic|tonicclonic|(?<!struvite\s)cluster(?!\sof)|petite?\s?mal|post\s?ictal|pre\s?ictal|grand\s?mal|eyes\sflickering|involuntary\smove|jerk|opisthotonus|(?<!coughing\s)(?<!sneezing\s)(?<!good\s)(?<!very\s)(?<!perfect\s)(?<!young\s)(?<!doesnt\s)(?<!no further\s)(?<!no more\s)(?<!bene)fits?(?!\sand</td></tr>
</table>

<table>
<tr><td></td><td>well?|\s\+\swell|\s&\swell?|\sfor|\sandw?|\sadn|\s?&|\sto|\sBC|\sin|\sdog|\syoung|\swell\scontrol)|emprosthotonus|absence(?!\s of)|muscular\shypertonic|myoclonic\sjerk|myoclonus</td></tr>
<tr><td>Hepatopathy</td><td>(?i)(?<=small\s)liver|liver(?=\samyloidosis|\sdamage|\sdegen|\sdisease|\sdisorder|\sdz|\sfail|\spath)|hepatic(?=\sdis|\sdz|\sinsuff|\scyto)|hepat(?=itis|opathy)|(?<!no\s)(?<!not\s)jaundice|\Walt\W?.?\s?\d{2,3}|alanine\saminotransferase\s?.?\s?\d{2,3}|(?<=elevated\s)bile\sacids?|(?<=high\s)bile\sacids?|(?<=increase\s)bile\sacids?|(?<=increased\s)bile\sacids?|(?<=increase\sin\s)bile\sacids?|bile\sacids?(?=\selevated|\shigh|\sincreased)|(?<=elevated\s)BA|(?<=high\s)BA|(?<=increase\s)BA\s|(?<=increased\s)BA|(?<=increase\sin\s)BA|BA(?=\selevated|\shigh|\sincreased)|samylin|denamarin|destolit|UCDA|ursodeoxycholic\sacid</td></tr>
<tr><td>Renal insufficiency</td><td>(?i)renal\sfailure|dilated\srenal\spelvis|renal\s(?=degen|dis|dys|dz|fail|insuff|.{1,9}dis|colic|comp)|(?<=enlarged) kidney|kidney\s(?=degen|dis|dys|dz|fail|insuff|.{1,9}dis|colic|comp)|(?<!poly)uria?c?\s|nephr\w{4,6}\s|\saki\s|acute\skidney\s injury|\sckd\s|chronic\skidney\sdisease|\scrf\s|chronic\srenal\sfailure|fanconi|papill?ary\snecrosis|specific\sgravity\s?=?-?\s?\d\.?\d+|u?s\.?g:?(?:\s?of)?\s?\(?(?:refr?act)?(?:ref)?(?:ometer)?\)?=?-?\s?:?\s?\.?\s?=?\s?\s?<?\s?\s?>?>?\s?\d+\.?\,?\d+|SDMA\s\d+|UPC\s\d{1,2}\.d|creat?\s?=?>?-?\s?\d+|creatinine\s\d+|\d\.?\d+\s?sg|sgrav\s\d+\.\d+|azota?emia|hypocalcaemi|ura?e?mia|renal\sdiet|ipakatine|pronefra</td></tr>
</table>

S1: The finalised regular expression for the four AEs of interest
